# Recent Progress in the Characterization, Synthesis, Delivery Procedures, Treatment Strategies, and Precision of Antimicrobial Peptides

**DOI:** 10.3390/ijms241411864

**Published:** 2023-07-24

**Authors:** Olalekan Olanrewaju Bakare, Arun Gokul, Lee-Ann Niekerk, Omolola Aina, Ademola Abiona, Adele Mariska Barker, Gerhard Basson, Mbukeni Nkomo, Laetitia Otomo, Marshall Keyster, Ashwil Klein

**Affiliations:** 1 Department of Biochemistry, Faculty of Basic Medical Sciences, Olabisi Onabanjo University, Sagamu 2002, Nigeria; swiftademon@gmail.com; 2 Department of Plant Sciences, Qwaqwa Campus, University of the Free State, Phuthadithjaba 9866, South Africa; gokula@ufs.ac.za (A.G.); 2017052495@ufs4life.ac.za (L.O.); 3 Environmental Biotechnology Laboratory, Department of Biotechnology, University of the Western Cape, Bellville 7535, South Africa; 3255882@myuwc.ac.za (L.-A.N.); 4176676@myuwc.ac.za (A.M.B.); 3373827@myuwc.ac.za (G.B.); mkeyster@uwc.ac.za (M.K.); 4 Plant Omics Laboratory, Department of Biotechnology, University of the Western Cape, Bellville 7535, South Africa; 4177137@myuwc.ac.za; 5 Department of Botany, H13 Botany Building, University of Zululand, Private Bag X1001, KwaDlangezwa 3886, South Africa; nkomom@unizulu.ac.za

**Keywords:** antimicrobial peptides, doping, coating, synthesis, characterization, broad-spectrum

## Abstract

Infectious diseases are constantly evolving to bypass antibiotics or create resistance against them. There is a piercing alarm for the need to improve the design of new effective antimicrobial agents such as antimicrobial peptides which are less prone to resistance and possess high sensitivity. This would guard public health in combating and overcoming stubborn pathogens and mitigate incurable diseases; however, the emergence of antimicrobial peptides’ shortcomings ranging from untimely degradation by enzymes to difficulty in the design against specific targets is a major bottleneck in achieving these objectives. This review is aimed at highlighting the recent progress in antimicrobial peptide development in the area of nanotechnology-based delivery, selectivity indices, synthesis and characterization, their doping and coating, and the shortfall of these approaches. This review will raise awareness of antimicrobial peptides as prospective therapeutic agents in the medical and pharmaceutical industries, such as the sensitive treatment of diseases and their utilization. The knowledge from this development would guide the future design of these novel peptides and allow the development of highly specific, sensitive, and accurate antimicrobial peptides to initiate treatment regimens in patients to enable them to have accommodating lifestyles.

## 1. Introduction

Antimicrobial peptides (AMPs) are small peptides that are highly ubiquitous and inhibit a wide range of bacteria, fungi, parasites, and viruses and have curative effects on cancer and other diseases [1]. These AMPs have been developed using preclinical and clinical strategies employing computational and experimental tools [2]. They are generally classified as antiviral, antibacterial, anti-inflammatory, antifungal, antiparasitic, and antitumor peptides. AMPs possess clear and compensatory advantages over conventional antibiotics such as slower occurrence of resistance, broad-spectrum antibiofilm functions, and the ability to regulate host immune systems [3]. The broad-spectrum function of AMP offers promising antimicrobial activities which would expand knowledge about the evolution of antimicrobial resistance. Systematic and comprehensive research has been conducted on AMPs’ classification, design methods, mechanism of action, and environmental factors affecting their activities; however, their delivery into tissues, cytotoxicity, and the bane of antimicrobial resistance remain elusive.

Multi-drug-resistant pathogens have constantly developed new strategies to bypass the immune system due to evolution, genetic plasticity, and mutational adaptation [4]. These “bypass” factors trigger specific responses for the pathogens to acquire genetic material or alter gene expression to produce resistance to almost all antibiotics in clinical practices [5]. The incidence of resistant pathogens is a major public health concern affecting the global population of humans because of the high mortality associated with them and the economic burden of multi-million dollars for developing sensitive molecules against them [6]. Therefore, the understanding of the biochemical and genetic basis of resistance would allow the development of novel molecules capable of little or no resistance by these multi-drug resistant pathogens. AMPs underscore the molecular design of molecules incapable of being resisted by pathogens due to their selective design and broad-spectrum activities [7]. Therefore, the emergence and popularity of AMPs in the revolution of medicine for complex medical approaches such as routine administration and its orthodox acceptance for commercialization and treating resistant pathogens is not surprising.

The delivery and formulation of AMPs have been reviewed comprehensively based on the interaction between AMPs and the host in the area of interaction and conjugation or modification [8]. Compared with small molecule antibiotics, which have undergone a century of development, peptide drugs have seen success only recently in the area of delivery and formulation. The formulation and delivery of AMPs are considered as important as the discovery of new AMP sequences [9]. The chemical structures of the AMPs can be modified to accommodate their deliverability [10]. Modifications have been done to enhance delivery through formulation systems such as encapsulation inside porous materials, attachment to the surface of materials, self-assembly, covalent conjugations, targeted delivery, and responsive release [11]. These delivery and formulation strategies, if not done properly, are technical hurdles such that clinical applications are not effective, largely due to the limitation of the stability and bioavailability of peptides.

Successful transfer of antimicrobial peptides from laboratory experiments to clinical practice for safe pharmacological formulations is a great property towards mitigating their cytotoxicity [2]. Cytotoxicity relates to the ability of a compound to exert a toxic effect on cells, or more specifically on human cells [12]. Antimicrobials are a family of medications that are particularly problematic in terms of cytotoxicity because their main function is to ultimately cause microbial cell death [13]. For instance, some antimicrobial peptides may be beneficial for preventing infected wounds at lower antimicrobially active concentrations; yet, at greater concentrations, they may demonstrate cytotoxicity that may negatively impact wound healing [14]. The development of antimicrobials in the future will therefore require cytotoxic profiling.

The present review aims to elucidate the recent advances of AMPs with high selectivity indices in terms of their delivery system, knowledge of the roles, cytotoxicity, and advances in the era of antibiotic resistance. These would help with the development of toxicity profiles of the AMPs to evaluate their relative activity against the cells of eukaryotic, mammalian, and human hosts, as well as their prospective usage in medical device applications. The awareness from this review would also reduce the risk of failure after validation from clinical trials. Although there is a low possibility that antibiotics will be discontinued during the development stage due to toxicity issues, withdrawals have still happened after clinical approval, leading to patient morbidity, death, and financial loss. For instance, temafloxacin was approved as an antibiotic in 1992 but was removed from the market six months later due to toxicity linked to hemolytic anemia, coagulation issues, and liver and kidney impairment [15]. The knowledge from this review would provide alternative antibiotics in AMP designs for sensitive treatment regimens and establish the best means to utilize and deliver them into tissues for precision medicine.

### 1.1. Antimicrobial Peptide Synthesis and Characterization 

AMPs play an important role in innate immunity in many species, serving as a first line of defense against invading pathogens [16]. There is growing interest in the development of AMPs as novel antimicrobial agents, due to their high specificity, low toxicity, and their compensatory advantage over antibiotics in the area of microbial resistance. AMPs are typically synthesized via solid-phase peptide synthesis (SPPS), a method developed by Bruce Merrifield in the 1960s [17]. SPPS involves the stepwise addition of amino acid building blocks to a resin-bound peptide chain, followed by cleavage of the peptide from the resin and purification by HPLC or other chromatography methods. One of the challenges in the synthesis of AMPs using this method is that many of these peptides contain amino acids, such as cysteine, tryptophan, and arginine, that can be difficult to incorporate using standard SPPS protocols [18]. Specialized protecting groups and coupling reagents may be required to overcome these challenges.

Another method employed in the synthesis of AMPs is the biological method which is very efficient and involves the use of recombinant techniques to produce them [19]. This method has been used to produce a 30–50 amino acid residue AMP such as defensin. However, this process has been found to have many drawbacks, including low production yields, an inability to remove the source’s inherent toxicity, and a failure to incorporate non-biological components. To address these issues, such peptides have been fused with thioredoxin, chloroplast expression systems, and other cell vectors [20]. This prevents cell lysis and promotes the creation of disulfide bonds. Another method for producing AMPs is known as enzymatic hydrolysis, which entails using one or more enzymes to hydrolyze a particular protein to produce short peptide sequences in the form of hydrolysates [21]. These peptides with antibacterial characteristics were created by hydrolyzing the protein found in *Salvia hispanica* seeds using alcalase and flavorzyme. Another approach is called “microbial fermentation”, which entails hydrolyzing a target protein using an enzyme secreted by a microbe to separate proteins into peptides for antimicrobial screening analysis. Because it is simpler to scale up and is more predictable within a short reaction time, enzymatic hydrolysis is preferred to the use of microorganisms to carry out the hydrolyzing process steps known as microbial fermentation [22].

Once synthesized, AMPs are typically characterized using a variety of techniques, including mass spectrometry, high-performance liquid chromatography (HPLC), and circular dichroism (CD) spectroscopy [23]. Mass spectrometry is a powerful tool for confirming the identity and purity of synthetic peptides, as well as for quantifying peptide concentration [24]. HPLC can be used to separate and purify the peptide from impurities, while CD spectroscopy can provide information about the secondary structure of the peptide [25]. In addition to these techniques, biological assays may be used to evaluate the antimicrobial activity of AMPs against specific bacterial or fungal strains [26]. These assays often involve exposing the microorganisms to the peptide and measuring the inhibition of growth or the killing of the cells. The synthesis and characterization of AMPs requires careful attention to both chemical and biological aspects of the peptide to produce a potent and effective antimicrobial agent.

### 1.2. AMPs in the Era of Antibiotic Resistance

Antibiotics are low molecular weight compounds that can either be produced by microorganisms, natural products, or synthetically, possessing antagonistic effects on the development of other microorganisms [27]. They either inhibit bacterial growth or completely kill bacteria by impairing the synthesis and function of the cytoplasmic membrane, impairing protein and nucleic acid synthesis, or inhibiting cell wall synthesis [28]. Since the discovery of the first antibiotic (penicillin) in the 1920s, several other antibiotics such as cephalosporins, chloramphenicol, tetracycline, glycopeptides, and amino glycopeptides have been developed [29]. These antibiotics have been reported to increase the expected lifespan and significantly reduce mortality rates in humans and animals [30]. For instance, the use of antibiotics increased the average lifespan in the US from 52 years in 1920 to 80 years in the 2000s [31]. Furthermore, the preventive administration of antibiotics to healthy children in communities with high child mortality rate increases their survival. Unfortunately, the enormous success achieved in the past decades has been jeopardized by excessive, uncontrolled, and overprescription of antibiotics, resulting in the development of bacterial strains resistant to antibiotic treatment [32].

Antibiotic resistance is a process in which bacteria evolve and develop resistance to drugs that were previously effective in treating infections [29]. It is one of the most serious threats to all aspects of life, including human health, plant health, and veterinary medicine [32]. Globally, there are approximately 2.8 million cases of antibiotic-resistant infections, resulting in 1.28 million deaths per year wasting more than 1.5 billion EUR due to treatment [33]. Furthermore, the UK antimicrobial resistance review recently reported that if antimicrobial resistance is not controlled, it will result in an estimated 10 million deaths and an economic loss of 100 trillion GBP annually by 2050 [34]. With the increasing rate of antibiotic resistance and the high cost of developing new drugs, there is an urgent need to develop alternatives to traditional antibiotics.

AMPs have sparked tremendous interest as potential future antibiotics [35]. They are naturally synthesized in cells of all living organisms, constituting a crucial part of the host’s innate defense mechanisms, which gives them several advantages over conventional antibiotics, including (1) the presence of short amino acid sequences makes them easier to synthesize, (2) they have multiple mechanisms of action making it difficult for bacteria to develop resistance to them, (3) they have a different mode of action from those of traditional antibiotics, (4) they kill bacteria rapidly since they are not affected by its resistance phenotype, (5) they are highly water soluble with good thermal stability [36,37]. The antibacterial activity of AMPs is enhanced by their cationic and amphiphilic (hydrophobic and hydrophilic) properties [1]. Generally, they have a net positive charge ranging from +2 to +9 due to the presence of high levels of basic amino acids such as lysine and arginine. They also have approximately 50% hydrophobic amino acids [38]. The cationic antimicrobial peptide forms electrostatic interactions with the anionic bacterial membrane, causing membrane disruption. Similarly, AMPs’ hydrophobic and hydrophilic properties increase their ability to interact with the lipid tails of the LPS and hydrophilic heads of phospholipids on the bacterial membrane. These interactions impair membrane permeability, resulting in metabolite leakage and bacterial cell death [39].

The structure of the bacterial membrane plays a major role in the mechanism of action of AMPs against bacterial cells. The membranes are composed of biomolecules such as lipopolysaccharides (LPS), phospholipids, peptidoglycans, and acidic polysaccharides that accord it a net negative charge [40]. These biomolecules differ in each bacterial strain. Gram-negative bacteria contain an impermeable outer membrane as a result of high levels of LPS, making them more antibiotic-resistant compared to Gram-positive bacteria. They also have an inner membrane made up of phospholipids [41]. In contrast to Gram-negative bacteria, Gram-positive bacteria lack an outer membrane but have thicker cell walls composed of peptidoglycans in which there are large amounts of teichoic acids (glycopolymers containing phosphodiester-linked polyol repeat units) that increase the negative charge on their surface [42]. AMPs primarily target bacteria cell membranes and adsorb to the negatively charged bacterial membrane via electrostatic interactions [39]. Adsorption to the bacterial surface enables them to permeate the outer membrane into the inner membrane via a complex series of interactions such as hydrophobic interactions, hydrogen bonds, and electrostatic interactions. These disrupt the electrochemical gradient, causing an influx of water into the cell, electrolyte leakage, and eventually cell lysis and death [43]. 

Three models (as previously described with diagrams by Bakare et al., (2021) [44] have been proposed to explain the membrane disruption activities of AMP; namely, the toroidal pore model involves the arrangement of AMPs perpendicularly to the membrane by interacting with the hydrophilic region and lipid bilayer, and then bending to form worm-like pores; the carpet-like model proposes a detergent-like disruption of the bacterial membrane caused by the parallel aggregation of peptides in a carpet-like manner around the bacterial membrane; and the barrel-stave model suggests accumulation of AMPs around the bacterial membrane in the form of a transmembrane pore, resulting in an outflow of the cytoplasm and subsequent collapse of the cell membrane [45,46]. Despite the enormous success of AMPs in killing bacteria, the following issues limit their full utilization: (i) they can also degrade the host cell membrane resulting in hemolysis, (ii) they are only stable at a specific pH, (iii) high production costs, (iv) tendency to be easily degraded by proteases, (v) the presence of metal ions in the cell, such as iron, inhibits their actions. Therefore, the factors mentioned above must be considered when designing AMPs to obtain the desired efficacy [46].

### 1.3. AMPs as Novel Antibacterial Agents with High Selectivity Indexes (SI)

The development of novel antibacterial agents with high selectivity indexes is an important area of research that holds great promise for the treatment of bacterial infections while minimizing the risk of toxicity and resistance development. The selectivity index is the ratio that measures the window between cytotoxicity and antibacterial activities [47]. A high selectivity index indicates that the drug is more selective for bacteria and has less toxicity to human cells [47]. High selectivity index AMPs are positively charged. They can distinguish between the anionic membrane of bacteria and the membrane of mammalian cells based on the different cell membrane compositions [48]. The development of new antibacterial peptides with strong selectivity indexes is an intricate and ongoing field of research. Achieving a high selectivity index is important to reduce toxicity and adverse effects on the host cells [49]. The objective is to design peptides that demonstrate remarkable selectivity for bacterial cells while causing negligible to no harm to human cells, offering a promising alternative to traditional antibiotics [50]. 

It takes a multidisciplinary approach that incorporates peptide chemistry, microbiology, structural biology, and computational approaches to discover and/or improve high-selectivity index peptides. Novel techniques such as isomerization, peptide lipidation, glycosylation, cyclization, other biomimetic terminal modifications, and multimerization can be used to engineer AMPs to increase their stability, activity, and targetability [51]. In a study by Mangmee et al., (2021) [48], six selected AMPs that had not previously been reported to exhibit anti-Salmonella activity were tested for their antibacterial ability against clinical Salmonella isolates, and the one (peptide Kn2-5R) that had potential action was employed as a template. The template was changed to produce four physicochemical variations, including D-enantiomerization, amidated C-terminal, enhanced positive charge, or increased hydrophobicity. They discovered a correlation between an increase in hydrophobicity and a decrease in SI. 

Timón et al., (2019) [52] demonstrated that an increase in the positive charge of AMPs enhances their selectivity index towards bacteria. The findings revealed that introducing lysine substitutions in the hydrophilic region of VmCT1, a peptide found in scorpion venom, led to an overall increase in positive charge. This increase resulted in higher antimicrobial activity values, ranging from 0.1 to 6.3 μmol L^−1^, compared to the original VmCT1 peptide, which ranged from 0.8 to 50 μmol L^−1^. Conversely, when lysine substitutions were made in the hydrophobic region of the helical structure, the antimicrobial activity values decreased. However, substituting lysine at the center of the hydrophobic face generated an analogue with antiplasmodial activity equivalent to that of VmCT1 (0.8 μmol L^−1^). This study demonstrated that by increasing the overall positive charge of VmCT1 peptides, it is possible to modulate their biological activities and decrease cytotoxicity. Furthermore, depending on the position of the added charge on the peptide, the AMP could display activity against different types of microorganisms which demonstrates that a single peptide template can be engineered to target various microorganisms.

Carratalá et al., (2020) [53] modified the terminal functional groups of the diphenylalanine (FF) peptide nanotube. Specifically, changing the terminal groups to amino (–NH_2_), carboxylic acid (–COOH), or both, and switching to D-isomers, resulted in an increase in the molecule’s ability to selectively target Gram-positive biofilm infection and in a decrease in its toxicity to mammalian cells. NH_2_-FF-COOH and NH_2_-ff-COOH nanotubes, contrary to NH_2_-FF-NH_2_, were able to selectively target bacterial cell membranes and penetrate the biofilm despite being neutrally charged. This study showed that altering AMPs’ terminal amino and carboxylic acid groups as well as their isomerization could enhance potency without the need to increase their positive charge.

Several approaches can be taken to develop novel antibacterial agents with high selectivity indexes. One approach is to target specific bacterial proteins or pathways that are essential for bacterial growth and survival but are not present in human cells [54]. This can be achieved through the use of high-throughput screening of compound libraries or rational drug design. Another approach is to modify existing antibacterial agents to improve their selectivity indexes [55]. This can be done by optimizing the pharmacokinetic and pharmacodynamic properties of the drug, such as its absorption, distribution, metabolism, and excretion (ADME), as well as its potency and spectrum of activity. In addition, the use of combination therapies can also improve selectivity indexes by targeting multiple bacterial pathways simultaneously, which can reduce the likelihood of resistance development and increase the efficacy of the treatment [55].

Recent advances in genomics and proteomics have also provided new opportunities for the discovery of novel antibacterial agents with high selectivity indexes. For example, the identification of new bacterial targets and the development of new screening technologies have led to the discovery of novel classes of antibiotics, such as lipopeptides and oxadiazoles [56]. Designing membrane-active peptide antibiotics with therapeutic potential presents a problem in that it is difficult to maintain effective antibacterial activity while minimizing unacceptable cytotoxicity for host cells. Any rational attempt to increase this selectivity must take into account the peptides’ mode of interaction with membranes and the factors that contribute to their capacity to differentiate between bacterial and eukaryotic cytoplasmic cells. Due to all of these factors, AMPs frequently exhibit high selectivity for bacterial over host cells and have a relatively low propensity to elicit resistance [57]. In addition to having significant pro-defensive effects on host immune cells, endogenously generated AMPs have the propensity to act on many targets in bacteria (Table 1 contains AMPs from databases such as APD3 with their codes (https://aps.unmc.edu/database/anti (accessed on 1 June 2023)) [58].

## 2. AMPs with Anti-Inflammatory Properties

Due to AMPs being involved in the first line of defense against pathogenic infection, they are often involved in antimicrobial and anti-inflammatory processes. Due to their ubiquitous nature in most living organisms as well as their therapeutic role, much research has been conducted on them [79]. Recent studies have been undertaken to produce AMP analogues to enhance the antimicrobial and anti-inflammatory properties of these peptides. Another study produced three analogues of the AMP CXCL14-C17 and the analogues were able to act on antibiotic-resistant bacteria and could have promising anti-inflammatory properties due to the inhibition of the tumor necrosis factor (TNF) and interleukin (IL-1β) [80]. Similarly, a study has been shown to produce two analogues (Mt6 and D-Mt6) of the AMP MAF-1 [81]. The analogues were observed to be stable and active against *A. baumannii* under many physiological settings as well as decreased TNF and IL-1β expression which suggests improved anti-inflammatory properties. Furthermore, D-Mt6 showed positive activity against lipopolysaccharide-induced inflammation through different mechanisms which included neutralizing lipopolysaccharide ability to produce inflammatory cytokines and suppressing the MAPK signaling. The aforementioned studies highlight the opportunity to improve existing AMPs to improve their anti-inflammatory properties instead of searching for new AMPs, which could become costly and labor-intensive. AMPs with similar properties as represented in the APD3 database (https://aps.unmc.edu/database/anti (accessed on 1 June 2023)) can be found in Table 1.

**Figure 1:**
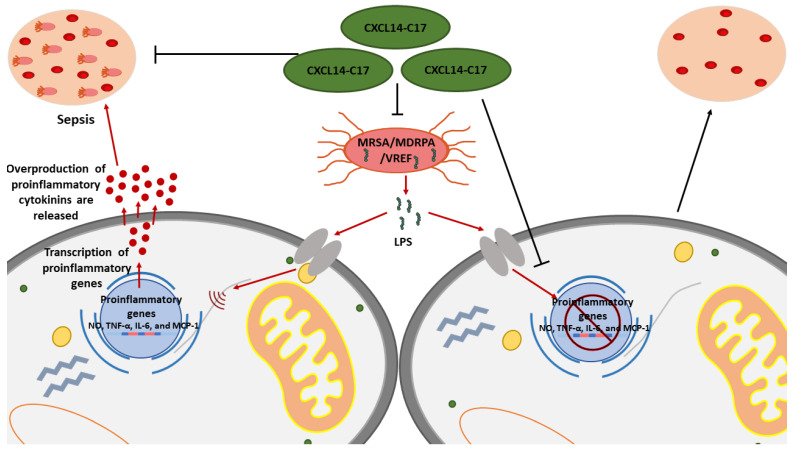
The anti-inflammatory mechanism of CXCL14-C17 on various antibiotic-resistant bacteria. Rajasekaran et al., (2019) [80], recorded that in lipopolysaccharide (LPS)-stimulated RAW264.7 cells the release of “bacterial LPS” instigated an induction of proinflammatory genes, such as the genes associated with nitric oxide (NO) production, tumor necrosis factor (TNF)-α, interleukin (IL)-6 and monocyte chemoattractant protein (MCP)-1. This induction of proinflammatory genes resulted in the overproduction of proinflammatory cytokinins ultimately leading to the sepsis of the RAW264.7 cells. However, the authors likewise depicted that with the presence of CXCL14-C17 AMP, in conjunction with the antimicrobial properties, these AMPs possess on bacteria (methicillin-resistant *Staphylococcus aureus* (MRSA), multidrug-resistant *Pseudomonas aeruginosa* (MDRPA), and vancomycin-resistant *Enterococcus faecium* (VREF)), the induction of proinflammatory genes was blocked, reducing the occurrence of sepsis. Thus, CXCL14-C17 illustrated both antimicrobial and anti-inflammatory properties against invading microbial infections.

When altering or producing these AMP analogues, proper strategies need to be considered to produce a peptide with enhanced properties, thus reducing the need for multiple iterations to obtain the enhanced properties desired. A study by Klubthawee and colleagues (2020) used a rational approach by using key structural physiochemical parameters to design novel hybrid peptides [82]. The peptide named PA-13 showed the best results due to its effectiveness across many parameters. Most importantly, the peptide inhibited the Toll-like receptor activation which led to the inhibition of the release of inflammatory cytokines and decreased further severe inflammation. Key considerations that should be noted when designing analogues of AMPs include the desired biological function of the AMP as well as the environment it will need to function in. Other AMPs with anti-inflammatory properties include citropin 1.1, cathelicidin 1, pexiganan acetate, jelleine 1, epinecidin 1, and porcin beta-defensin 2, to mention just a few (Figure 1). Utilizing computational techniques for database retrieval, AMPs have been found to have broad-spectrum antibacterial and anti-inflammatory actions as well as well-described physicochemical features that are effective against illnesses [83].

### 2.1. Role of AMPs in Medicine

Multi-drug-resistant pathogens represent a considerable risk and dynamic issue to both patients and society, thus posing significant global health and economic burden [84]. It is vital to find new medicines with novel mechanisms of action that are less likely to cause bacterial resistance and thus give rise to the growing problem of antibiotic resistance. AMPs, sometimes referred to as host defense peptides, have recently attracted a lot of attention as potential next-generation antibiotics [85,86]. As a result, research has shown that AMPs are generated from both natural and synthetic sources, emphasizing their ability to not only kill resistant microbial pathogens, but also proposed other benefits such as wound healing, anti-tumor, and regulating of the immune system [87]. Here, we review literature describing the involvement of AMPs in advancing research in medicine and evaluating their properties in antibacterial activity followed by their application in some infectious diseases.

Antibacterial peptides have long shown significant promise in medicine, dating back to 1939, when René Dubos discovered gramicidin, the first clinically tested antibacterial agent that has been reported to protect mice against pneumococcal disease [88]. The discovery of gramicidin from a soil Bacillus strain is believed to have helped revive the stalled interest in penicillin and launched the era of antibiotics. Following René Dubos’ discovery, multiple AMPs were isolated and characterized from various species, including both prokaryotic and eukaryotic kingdoms, with most AMPs exhibiting broad-spectrum antimicrobial action against bacteria, fungi, and viruses [89]. While the first advancement in identifying human-derived AMPs came from Zeya and Spitznagel [90], they did so by identifying cationic proteins contained in leukocyte lysosomes. Their protein exhibited broad-spectrum antibacterial activity against several microorganisms, suggesting that it can combat a wide range of infections. This demonstrated selectivity for certain bacteria, hinting that personalized therapies for diverse illnesses are possible [91]. AMPs are still being discovered to date, with over 3000 human AMPs discovered in the Antimicrobial Peptide Database (APD3) (https://aps.unmc.edu/database/anti (accessed on 1 June 2023)). The introduction of the APD3 database greatly advanced the field of AMP research as it was able to provide a consolidated and curated database of AMPs [92].

The availability of such a large database improves knowledge and prospective applications of AMPs, including their investigation of AMPs’ physicochemical parameters, potential efficacy against SARS-CoV and other infectious illnesses, as well as insights into their mechanism of action and structure–activity connections. It is important to note that AMP identification and characterization is a continuous research area, that was further advanced during the SARS-CoV outbreaks, as most researchers were looking for novel antiviral medications to prevent COVID-19 sickness [93].

Other research work further advanced AMPs research by providing a protein peptide database that includes AMPs and their interactions with various proteins [94]. The significant findings in the Pirtskhalava et al., (2021) [94] study lie in the development of a protein peptide database that includes AMPs and their interactions with other proteins. The mention of AMPs targeting SARS-CoV supports the possible use of AMPs as therapeutic candidates against SARS-CoV. While research on AMPs and COVID-19 showed promising results, it is crucial to remember that they are still in the early phases, and further research, including preclinical and clinical trials, is needed to examine their effectiveness, safety, and potential for clinical use against SARS-CoV-2. This would aid in determining whether the AMPs used have a comparable impact on colistin, an antibacterial drug that is effective in combating multidrug-resistant bacterial infections [95]. It is now being used as a last-resort therapy for multidrug-resistant bacterial infections, particularly due to the higher risk that involves its side effects which include kidney toxicity, neurotoxicity, and respiratory complications.

### 2.2. Nanotechnology-Based Delivery Systems for AMPs

Nanotechnology is the process of modifying matter at a size close to the atomic level to create novel structures, materials, and gadgets [96]. The technique offers advances in science across a wide range of industries, including manufacturing, consumer goods, energy, and medicine [97]. Nanotechnology makes it possible to avoid first-pass metabolism by delivering medications that are not particularly water-soluble [98]. Nanotechnology allows AMPs to undergo absorptive endocytosis, confers the ability to stay in the blood circulation for an extended period, and increases the oral bioavailability of drugs [99]. This results in less fluctuating plasma levels and minimized side effects. Nanotechnology has emerged as a promising approach for improving the efficacy and safety of AMPs [100]. These systems involve the use of nanoparticles, liposomes, or other nanoscale carriers to deliver AMPs to their target sites [101]. One advantage of nanotechnology-based delivery systems is that they can protect AMPs from degradation by enzymes and other factors in the body, which can improve their stability and prolong their activity [102]. In addition, these systems can enhance the bioavailability of AMPs by improving their absorption and distribution in the body.

Nanoparticles, such as polymeric nanoparticles, lipid nanoparticles, and metal nanoparticles, have been used to deliver AMPs to their target sites [103]. These nanoparticles can be designed to have specific properties, such as size, surface charge, and surface chemistry, that can affect their interactions with cells and tissues [104]. Liposomes, which are spherical structures composed of a lipid bilayer, have also been used to deliver AMPs. Liposomes can encapsulate AMPs within their aqueous core or lipid bilayer, providing protection from degradation and enhancing their bioavailability [105]. In addition, other nanoscale carriers, such as dendrimers and carbon nanotubes, have been explored for the delivery of AMPs [106]. These carriers can also be designed to have specific properties that can improve their interactions with cells and tissues. Nanotechnology-based delivery systems represent an exciting area of research that holds great promise for the development of more effective and targeted antimicrobial therapies [107]. This would enable a sensitive and precise screening technique for AMP development against infections or the treatment of diseases in patients for early treatment regimens before or after the onset of symptoms, allowing them to adopt adapting lifestyles.

## 3. AMP-Doped and AMP-Releasing Biomaterials and Coating

AMPs are a promising class of natural antibiotics that can be used to combat infections. One strategy for enhancing the activity of AMPs is to incorporate them into biomaterials or coatings or to use these materials as a platform for the controlled release of AMPs [108]. AMP-doped biomaterials involve the incorporation of AMPs into a polymer matrix or other material, such as hydrogels, nanoparticles, or films [109]. This can be achieved through various techniques, such as electrospinning, self-assembly, or covalent coupling. AMP-doped biomaterials have been shown to have excellent antimicrobial activity against a variety of pathogenic organisms, including drug-resistant strains [110]. AMP-releasing biomaterials involve the controlled release of AMPs from a polymer matrix or other material. This can be achieved through various mechanisms, such as diffusion, degradation, or stimuli-responsive release. AMP-releasing biomaterials have the advantage of providing sustained antimicrobial activity over an extended period while minimizing the risk of toxicity and resistance development [111].

Coatings are another approach for incorporating AMPs into biomaterials which can be applied to the surface of medical devices, implants, or other materials to provide a barrier against bacterial colonization and infection [112]. AMP-based coatings are highly effective against a variety of bacteria, including those that are resistant to conventional antibiotics. These materials slowly release the peptides over time, providing a sustained antimicrobial effect [102]. This approach has shown promise in reducing the risk of implant-related infections and other medical device-related infections. On the other hand, AMPs releasing coatings can be applied to the surface of medical devices or implants to provide a rapid antimicrobial effect upon contact with pathogens [108]. These coatings can be designed to provide broad-spectrum protection against a range of bacteria and viruses. AMP-based biomaterials and coatings offer a promising solution to prevent infections and improve health outcomes which require constant optimization in their design and application for different medical devices and coatings.

### 3.1. AMPS and Mesenchymal Stem Cells

Mesenchymal stromal cells or stem cells (MSCs) are plastic adhering spindle-shaped cells that can be obtained from adipose tissue, placenta, bone marrow, and various other perivascular niches. These cells have been reported to possess traits similar to stem cells due to their differentiation and regeneration abilities [113]. MSCs have also gained a lot of attention in cancer research, being linked to tumor development. It has been reported that MSCs can locate and alter the behavior of tumors and either act as a promoter or suppressor of cancer cells [114]. Additionally, these cells have also been shown to regulate the activity of immune cells involved in tumor regression. One of the mechanisms used by MSCs to exhibit their influence on cells within their environment includes the production of AMPs [115].

This has piqued the interest of researchers that these cells-containing AMPs have biological flexibility (cellular differentiation capacity) and therefore to an extent, metabolic flexibility in terms of their gene products. In a study by Haman and colleagues, the authors reported the depolarization of the cellular membranes of *E. coli* and *S. aureus* in response to *Equine*-derived AMPs [116], thereby highlighting the potential of these AMPs in treating bacterial-associated skin infections. Additionally, a human bone marrow-derived AMP, cathelicidin- LL-37, had an inhibitory effect on the growth of several bacterial species namely *E. coli*, *P. aeruginosa,* and *S. aureus* [117]. Hepcidin 25 (hep-25), an AMP produced by hepatocytes and MSCs, has also been shown to have strong bactericidal activity against several strains of *P. aeruginosa, Stenotrophomonas maltophilia, Enterococcus faecium, Staphylococcus. aureus,* and *Staphylococcus epidermidis*, some of which are multidrug-resistant [118]. Furthermore, neutrophil gelatinase-associated lipocalin (NGAL) byproducts produced by bone marrow MSCs (BMSC) have been shown to bind with metalloproteins such as bacterial siderophores. This binding contributes to strong antibacterial activity as the bacteria are unable to obtain nutrients [119]. These studies highlight the major potential of MSCs and their derived AMPs for future advancements in medicine.

However, there are some limitations associated with MSCs and therefore the AMPs they produce. Studies have shown that in vitro isolation of MSCs does not necessarily present a completely homogenous population of stem cells [120]. It could be proposed that in their microenvironment, MSCs are in the presence of other cellular populations. In this environment, there are fluctuations in the presence of proteases, micro RNAs, intracellular conditions, cell-to-cell signaling, and hypoxia [121]. These environmental factors may influence the future capabilities of MSCs as in biology, the environment often drives genetic selection. This may affect the phenotypic characteristics of matured MSCs. The variation in the characteristics and differential potential of MSCs from different mammalian tissues further support the notion of micro-environmentally driven MSC phenotypic potential [122]. Therefore, more attention needs to be drawn to obtaining homogenous populations of MSCs as well as linking the characterization and differential capacities to the source of the AMPs.

The use of single nucleotide polymorphisms (SNP)-genotyping for the identification of environmentally driven variation between MSCs has been well described in genetics. The use of this approach has proven useful in the field of precision medicine for understanding the contribution of genetic variation to altered phenotypic pathobiology and response to treatments [123]. These polymorphisms between MSCs may provide insight into the future application of those cells. Furthermore, coupling these outputs with transcriptomics and proteomic research could evaluate the contribution of the aforementioned variation to functional genetic products. These may be used as future molecular markers for the selection of MSCs with an intended application [124]. Additionally, given that these omics technologies are moving towards single-celled approaches, future studies should investigate heterogeneity amongst supposed homogeneous populations of MSC cells. In their study, a single-celled multi-omics approach was used to identify transcripts and proteins involved in the differentiation of homogeneous monocytes differentiate into macrophage-like cells [125]. Using these approaches, comprehensive application of MSCs as well as their AMPs can be drawn potentially on a single cell/population basis in response to an experimental environment.

### 3.2. AMPs and Biofilms

Biofilms refer to communities of microorganisms that either adhere to surfaces or are suspended in liquids such as flocs [126]. They are surrounded by a self-produced extra polymeric substance (EPS) that is composed of polysaccharides, proteins, glycoproteins, glycolipids, metabolites, and extracellular DNA (eDNA) [127,128]. The development of biofilms begins with the initial attachment of planktonic bacteria to surfaces and the formation of microcolonies. Following adhesion, EPS synthesis starts, which leads to the early formation of the biofilm architecture. The glycopeptides, glycolipids, and lipopolysaccharides found within the EPS help to keep biofilms intact [128]. Maturation of biofilm architecture, and the dispersal of single cells from mature biofilms, can initiate the cycle again [127].

The lifestyle of a bacterium embedded in a biofilm differs remarkably from its free-living counterparts [129]. Biofilms serve as a survival strategy developed by prokaryotes to respond to environmental stresses such as predator attacks, chemical treatments, nutrient limitations, oxidative stress, hypoxia, and drought. This difference can be observed in terms of the population characteristics of internal cells, physical and chemical properties of the extracellular matrix (ECM), and intercellular interactions [130]. They are not only essential for the survival of bacteria but also have a wide range of impacts on human health.

It is widely acknowledged that microorganisms living within biofilms are less susceptible to antimicrobial agents and host immune defenses [131]. Bacteria embedded in these bacterial communities often have a 1000-fold increased tolerance to antibiotics [132]. Consequently, diseases caused by biofilms pose significant challenges in terms of treatment. Roughly 65–80% of infectious diseases are estimated to be associated with biofilms [133]. Medical device-associated infections, bacterial contaminations during food processing and storage, and biofouling in industrial production mainly originate from the failure of bacterial biofilm eradication. Most antibiofilm agents available to date have been antimicrobial agents with bactericidal effects, which are ineffective for treating biofilm-related infections and can lead to microbial resistance when used for a long time [134]. Hence, it is important to design or screen novel antibiofilm agents that can effectively prevent biofilm formation or eradicate existing biofilms. In recent years, there has been growing interest in exploring alternative strategies to combat biofilm-associated infections [135]. The results of these studies have led to AMPs being considered as an alternative drug to conventional antibiotics [54].

Natural occurring or synthetic AMPs have been shown to prevent the formation of biofilms and eradicate already existing ones [136]. There are two main targets for the disruption of biofilm formation: dispersal of the biofilm EPS or the elimination of the bacteria that reside within the EPS. Typically, the effectiveness of antibiotics in eradicating bacteria embedded in a biofilm is greatly diminished, often requiring 1000-fold concentrations because the antibiotics cannot translocate into the EPS and therefore do not reach the bacterial cells. Conversely, AMPs offer potential as anti-biofilm agents due to their diverse mechanisms, such as disrupting cell membranes, inhibiting protein function, binding with DNA, and neutralizing polysaccharides such as lipopolysaccharide and lipoteichoic acid [128]. Certain AMPs can traverse the biofilm EPS via pores or openings within the lipid component, while others can disperse biofilms. Examples of such peptides include temporin B and indolicidin that are capable of traversing the pores of lipid membranes and citropin capable of dispersing biofilms.

AMPs possess inherent properties that make them effective against biofilms. Research has shown the ability of AMPs to disrupt biofilm formation, inhibit biofilm growth, and eradicate preformed biofilms [137]. AMPs can penetrate the biofilm matrix, interact with microbial cells, and exert their antimicrobial effects through multiple mechanisms. These mechanisms include membrane disruption, pore formation, inhibition of biofilm matrix production, and modulation of microbial signaling pathways. The multifunctional nature of AMPs enables them to target various components of biofilms, making them effective against a wide range of pathogens. Combining AMPs with conventional antimicrobial agents has shown promising results in combating biofilm-associated infections [138]. The synergistic interactions between AMPs and antibiotics can enhance the overall antimicrobial activity and overcome the resistance mechanisms of biofilms. AMPs can potentiate the activity of antibiotics by permeabilizing the biofilm matrix, enabling better penetration of the antimicrobial agent. Additionally, some studies have reported that AMPs can restore the susceptibility of antibiotic-resistant biofilms, making them susceptible to conventional therapy [139,140].

Advancements in peptide engineering and design have facilitated the development of novel AMPs with enhanced efficacy against biofilms [2]. Rational design strategies, such as sequence modification, incorporation of non-natural amino acids, and optimization of physicochemical properties, have been employed to improve the antimicrobial activity, stability, and selectivity of AMPs. Furthermore, the combination of AMPs with other biofilm-disrupting agents, such as enzymes and nanoparticles, has shown synergistic effects in biofilm eradication. The therapeutic potential of AMPs against biofilm infections has been investigated in various in vivo models [138,141]. These studies have demonstrated the efficacy of AMPs in reducing biofilm burden, preventing biofilm formation, and promoting wound healing. Moreover, some AMPs have successfully progressed to clinical trials, highlighting their potential as future therapeutic options for biofilm-associated infections [142]. However, challenges such as high manufacturing costs, limited stability, and potential cytotoxicity need to be addressed for the clinical translation of AMPs.

### 3.3. Cytotoxicity of AMPs

Over many years, chemotherapy was shown to be the most influential and most effective in the treatment of cancer, yet the drugs commonly employed were unable to discern healthy cells from cancerous cells, commonly resulting in the damage of healthy cells and resulting in major side effects [143]. Hence, the investigation into the potential use of AMPs has been gaining popularity in the treatments of cancer and bacterial infections. However, there have been major limitations accompanying the clinical application of AMPs [144]. One of the leading limitations restricting the implementation of AMPs into clinical use is the cytotoxic nature of AMPs towards not only cancerous cells but also healthy cells, hence their capacity to discern cancerous cells from healthy cells proposed a similar problem that current chemotherapy drugs presented. Therefore, the major topics of AMPs research has been focused on the discovery and development of a multitude of AMPs which has increased capabilities to contest a broader spectrum of bacterial and cancerous cell membranes [145,146].

Membranes of bacterial pathogens and cancerous cells have been key targets for AMP research, and recent studies have shown that to perform as a broad-spectrum AMP and to achieve AMPs with a higher affinity to these membranes, the peptide sequences of AMPs should be augmented [145]. In a study by a group of authors, it was proposed that partial D-amino acid substitution at the protease cleavage site of AMPs might be vital in not only reducing the cytotoxicity of AMPs to healthy cells by improving the potency of the AMP’s antimicrobial properties but also improving their proteolytic stability [146]. A study adopted this proposed idea and evaluated the potential of this approach in reducing the cytotoxicity of a clinical AMP, WLBU2, towards the host cell. These authors developed a novel engineered cationic AMP (eCAP) of WLBU2, by implementing a D-enantiomerization approach (a substitution of L-WLBU2 with D-WLBU2) [147]. It was noted that this novel form of D-WLBU2 exhibited a broad activity against multidrug-resistant (MDR) bacteria. In the majority of the cases of AMP research, the in vivo data are typically not investigated, hence Di et al. (2020) [147] deemed it important to determine whether D-enantiomerization of WLBU2 in vitro activities was translatable to in vivo efficacy. The authors recorded that coupled with broad antimicrobial activity, the novel D-WLBU2 exhibited a reduction in toxicity and increased stability in isolated human erythrocytes and blood leucocytes (both were included to observe the effects of D-WLBU2 on both nucleated and nonnucleated cells). The study thus indicated that the proposed approach of D-enantiomerization of WLBU2 successfully achieved reduced host toxicity while simultaneously reducing MDR-induced respiratory infection. In a different study by Beaudoin et al. (2018) [148], these authors took the approach of designing a synthetic cationic AMP (CAP), by generating numerous peptides which had a diverse array of charged patterning. The authors indicated that these alterations increased the amphipathic nature of 6K-F17, in such a way that it imitated the design of natural CAPs [148]. In addition, the synthetic CAP displayed high antimicrobial activity against *P. aeruginosa* infection and no toxicity to mammalian cells. On the other hand, Hou et al., (2022) [143], focusing on oral squamous cell carcinoma (OSCC), demonstrated that another cationic AMP, NRC-03, exhibited a potent and selective cytotoxicity towards negatively charged cancerous cells, by inducing mitochondrial oxidative stress-mediated altered mitochondrial function and apoptosis in OSCC cells.

The previous studies mentioned above revolved around developing and designing AMPs with enhanced selectivity towards negatively charged harmful cells; however, the mechanism by which these AMPs induced cytotoxicity in these cells was not elucidated. Gong et al., (2019) [145] demonstrated that cationic AMPs destroyed pathogenic bacteria (Gram-negative and Gram-positive) utilizing inducing membrane structural damage resulting in cytoplasmic leakage. The authors demonstrated, by using neutron reflection (NR), that cationic AMPs were more damaging to anionic and unsaturated phospholipids. It was concluded that the more anionic phospholipids bacterial pathogens possess, the more susceptible they were to cationic AMPs. The study by Hou et al., (2022) [143] elucidated the direct effects of cationic AMPs on negatively charged cells. The authors illustrated that NRC induced cancer cell death by targeting the mitochondria (an important organelle in tumour initiation and progression). These NRC-03 AMPs were shown to enter the OSCC cells and attach to the OSCC mitochondria. Here, these AMPs then disrupted the cyclophilin-dependent (CypD) regulation of the mitochondrial channel (mPTP), which led to the constant opening of the mPTP channel, releasing an increasing amount of reactive oxygen species (ROS) into the cytosol of the OSCC cells. Hence, it was concluded that NRC-03′s mode of inducing cytotoxicity in OSCC cells was through inducing CypD-mPTP axis-mediated mitochondrial oxidative stress, leading to apoptosis and cell death.

**Figure 2:**
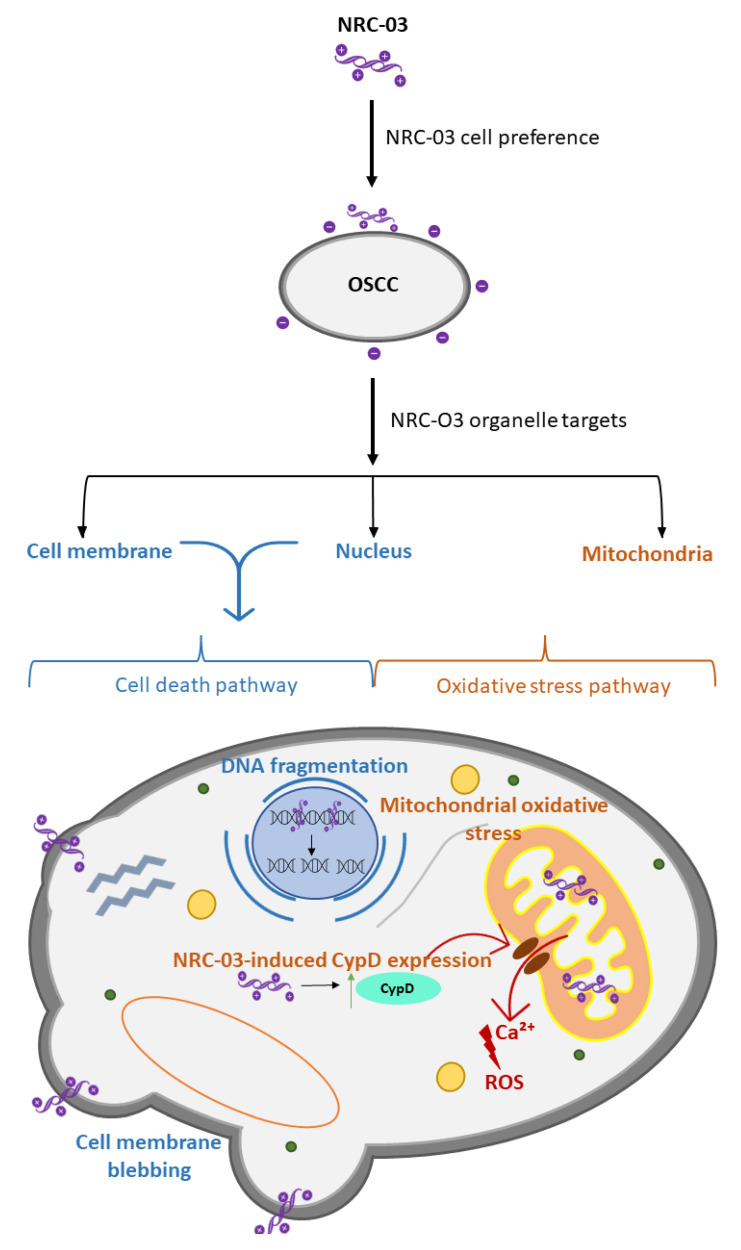
Representation of the molecular mechanisms of NRC-03-induced apoptosis and cell death in oral squamous cell carcinoma (OSCC) cells. The cationic nature of NRC-03 grants this cationic AMP to purposefully target negatively charged cancer cells, such as OSCC. NRC-03 easily enters the cancerous cells and has three main targets within these cells. They were shown to be bound to the cell membrane, nucleus, and mitochondria. Two pathways thus ensue wherein the cell death/apoptotic pathway NRC-03 bound to the nucleus results in DNA fragmentation while the cell-membrane-bound NRC-03 has been shown to increase the prevalence of blebbing. More importantly, Hou et al., (2022) [143], noted that NRC-03-induced expression of CypD has been shown to cause the prolonged opening of the mPTP channel, which leads to the increased efflux of calcium (Ca^2+^) and Reactive Oxygen Species (ROS) into the cytosol. This prolonged CypD-dependent opening of the mPTP channel thus caused mitochondrial dysfunction, swelling, and rupture, supplementing cell death in the OSCC cells. Hence, Hou et al., (2022) [143], stated that the molecular mechanism of NRC-03-induced cell death is mainly caused by the mitochondrial oxidative stress-mediated altered mitochondrial function.

The selective cytotoxicity of a cationic AMP toward the OSCC cells described by Hou et al., (2022) [143] together with the abovementioned studies involves either the development or designing of cationic AMPs (Figure 2). These studies reveal that the majority of AMPs enhanced selective cytotoxicity (ability to differentiate between normal cells and either bacterial pathogen or cancerous cells) which revolves around AMPs’ charged state. AMPs normally recognize their targets through electrostatic interactions [149]. Cancerous cells with a majority of phosphatidylserines on their surfaces, and pathogenic bacterial membranes (Gram-negatives comprising of lipopolysaccharides and Gram-positives comprising of lipoteichoic acid) all present a highly negatively charged surface, compared to normal healthy host cells, and due to their (bacterial pathogens and cancerous cells) high density of electronegative charges, cationic AMPs (positively charged surfaces) exhibit a strong attraction and interaction to these cells [150]. Hence, there is a need for increasing the capacity to differentiate between these unwanted cells and the host’s healthy cells. Furthermore, because these interactions are not mediated by specific receptors, the D-enantiomerization of AMPs does not interrupt the binding capacity of AMPs. Hence, cationic AMPs have paved the way to decrease host cytotoxicity while simultaneously maintaining the effectiveness of AMPs’ cytotoxic nature toward cancerous cells and multidrug-resistant bacterial pathogens.

## 4. Conclusions

The importance of AMPs is enormous due to their compensatory advantages over conventional antibiotics. These benefits show the need to improve the design of these novel peptides. The present review holistically approached this by taking cognizance of the recent development of AMPs in terms of synthesis, characterization, benefits, cytotoxicity, coating, and doping, as well as their delivery into tissues. This would allow adequate protection against microorganisms and disease transmission for effective and sensitive inhibition of their growth. Additionally, the search for new treatments for human illness, elimination of the spread of pests, and ease of delivery all benefit from using AMPs. To address a variety of issues relating to pathogen resistance and the lack of sensitivity of antibiotics, these underutilized AMPs and their products hold enormous promise as a novel source of drug development for treating human infections and other disorders.

## Figures and Tables

**Table 1:** Novel antimicrobial peptides (AMPs) with anti-inflammatory functions.

AMP Database Codes	Specific Names	Sequences	Specific Functions	References
AP00351	Citropin 1.1	GLFDVIKKVASVIGGL	Peptidoglycan breakdown for its access to the cytoplasmic membrane	[59]
AP00557	Cathelicidin 1	RVKRVWPLVIRTVIAGYNLYRAIKKK	Bind and disrupt negatively charged membranes resulting in cell death	[60]
AP00952	Pexiganan acetate	GIGKFLKKAKKFGKAFVKILKK	Disturbance/disruption of the cell membrane permeability	[61]
AP01210	Jelleine-I	PFKLSLHL	Improvement of intestinal mucosa tight junction	[62]
AP01328	Epinecidin-1	GFIFHIIKGLFHAGKMIHGLV	Immunomodulatory and wound-healing functions	[63]
AP01372	CXCL14	SKCKCSRKGPKIRYSDVKKLEMKPKYPHCEEKMVIITTKSVSRYRGQEHCLHPKLQSTKRFIKWYNAWNEKRRVYEE	Inhibition of colorectal cancer migration, invasion, and epithelial-to-mesenchymal transition (EMT) by suppressing NF-kappaB signaling	[64]
AP01550	Dybowskin-2CDYa	SAVGRHGRRFGLRKHRKH	Promotion of cell proliferation	[65]
AP01590	porcine beta-defensin 2	DHYICAKKGGTCNFSPCPLFNRIEGTCYSGKAKCCIR	Alleviation of inflammation via interference with the TLR4/NF-κB Pathway	[66]
AP01751	Papiliocin	RWKIFKKIEKVGRNVRDGIIKAGPAVAVVGQAATVVK	Possession of broad-spectrum antibacterial activities	[67]
AP01794	Temporin-1CEa	FVDLKKIANIINSIF	Possession of broad-spectrum antibacterial activities	[68]
AP01976	Coprisin	VTCDVLSFEAKGIAVNHSACALHCIALRKKGGSCQNGVCVCRN	Prevention of *Clostridium difficile*-mediated acute inflammation and mucosal damage through selective antimicrobial activity	[69]
AP02095	secretory leukocyte protease inhibitor	SGKSFKAGVCPPKKSAQCLRYKKPECQSDWQCPGKKRCCPDTCGIKCLDPVDTPNPTRRKPGKCPVTYGQCLMLNPPNFCEMDGQCKRDLKCCMGMCGKSCVSPVKA	Protection of tissues by inhibiting the proteases, such as cathepsin G, elastase, and trypsin from neutrophils; chymotrypsin and trypsin from pancreatic acinar cells; and chymase and tryptase from mast cells	[70]
AP02202	Cathelicidin-PY	RKCNFLCKLKEKLRTVITSHIDKVLRPQG	Inhibition of the activation of Toll-like receptor 4 (TLR4) inflammatory response pathways induced by lipopolysaccharide (LPS)	[71]
AP02569	Hc-CATH	KFFKRLLKSVRRAVKKFRKKPRLIGLSTLL	Hc-cath exerts an antimicrobial effect against *P. aeruginosa* and *S. aureus* and promotes Galleria survival	[72]
AP02570	GL13K	GKIIKLKASLKLL	GL13K exhibits bactericidal activity against planktonic bacteria and low toxicity against mammalian cells	[73]
AP02621	SibaCec	GKLTKDKLKRGAKKALNVASKVAPIVAAGASIAR	SibaCec can function as an immune regulator, inhibiting the host defense system against infection	[74]
AP02626	Cl-CATH2	LIQRGRFGRFLGRIRRFRPRINFDIRARGSIRLG	Cl-CATH2 exerts broad-spectrum but moderate antimicrobial activities	[75]
AP02629	dCATH	KRFWQLVPLAIKIYRAWKRR	Activation of autophagy, which enhances antimicrobial effects against diverse pathogens	[76]
AP02703	Periplanetasin-5	MKTFLRLYRSLINKVLH	Control of inflammation by inhibiting phosphorylation of MAPKs, and reduction of degradation of IκB.	[77]
AP02901	Cathelicidin-PP	ASENGKCNLLCLVKKKLRAVGNVIKTVVGKIA	Reduction of bacterial load in clinically relevant infection models	[78]

## Data Availability

Not applicable.
